# Relationship between Respiratory Microbiome and Systemic Inflammatory Markers in COPD: A Pilot Study

**DOI:** 10.3390/ijms25158467

**Published:** 2024-08-02

**Authors:** Carme Casadevall, Sara Quero, Laura Millares, Rosa Faner, Borja G. Cosío, Germán Peces-Barba, Ady Castro-Acosta, Concepción Montón, Alexandre Palou, Sergi Pascual-Guardia, Alvar Agustí, Joaquim Gea, Eduard Monsó, on behalf of the BIOMEPOC group

**Affiliations:** 1 Hospital del Mar Research Institute (IMIM), Parc de Recerca Biomèdica de Barcelona (PRBB), 08003 Barcelona, Spain; spascual@psmar.cat (S.P.-G.); jgea@psmar.cat (J.G.); 2 Centro de Investigación Biomédica en Red, Área de Enfermedades Respiratorias (CIBERES), Instituto de Salud Carlos III, 28029 Madrid, Spain; squero@igtp.cat (S.Q.); rfaner@recerca.clinic.cat (R.F.); borja.cosio@ssib.es (B.G.C.); gpeces@quironsalud.es (G.P.-B.); alvar.agusti@clinic.cat (A.A.); 3 Department of Medicine and Life Sciences (MELIS), Universitat Pompeu Fabra (UPF), 08003 Barcelona, Spain; 4 Airway Inflammation Research Group, Parc Taulí Research and Innovation Institute-I3PT–Parc Taulí Foundation, 08208 Sabadell, Spain; lmillares@igtp.cat (L.M.); cmonton@tauli.cat (C.M.); emonso@tauli.cat (E.M.); 5 Catalan Institute of Oncology–ICO, Hospitalet de Llobregat, 08908 Barcelona, Spain; 6 Servei de Pneumologia (Institut Clínic de Respiratori) and Dispositiu Transversal d’Hospitalització a Domicili (Direcció Mèdica i d’Infermeria), Hospital Clínic–Fundació Clínic per la Recerca Biomèdica, Universitat de Barcelona, Institut d’Investigacions Biomèdiques August Pi i Sunyer (IDIBAPS), 08036 Barcelona, Spain; 7 Servei de Pneumologia, Hospital Son Espases–Institut d’Investigació Sanitària de Palma (IdISBa), 07120 Palma de Mallorca, Spain; alexandre.palou@ssib.es; 8 Servicio de Neumología, Fundación Jiménez Díaz, Universidad Autónoma de Madrid, 28049 Madrid, Spain; 9 Servicio de Neumología, Hospital 12 de Octubre, 28041 Madrid, Spain; ady@h12o.es; 10 Servei de Pneumologia, Hospital Universitari Parc Taulí, 08208 Sabadell, Spain; 11 Servei de Pneumologia, Hospital del Mar, 08003 Barcelona, Spain

**Keywords:** COPD, respiratory microbiome, systemic inflammation, frequent exacerbators, eosinophils

## Abstract

The respiratory microbiome may influence the development and progression of COPD by modulating local immune and inflammatory events. We aimed to investigate whether relative changes in respiratory bacterial abundance are also associated with systemic inflammation, and explore their relationship with the main clinical COPD phenotypes. Multiplex analysis of inflammatory markers and transcript eosinophil-related markers were analyzed on peripheral blood in a cohort of stable COPD patients (n = 72). Respiratory microbiome composition was analyzed by 16S rRNA microbial sequencing on spontaneous sputum. Spearman correlations were applied to test the relationship between the microbiome composition and systemic inflammation. The concentration of the plasma IL-8 showed an inverted correlation with the relative abundance of 17 bacterial genera in the whole COPD cohort. COPD patients categorized as eosinophilic showed positive relationships with blood eosinophil markers and inversely correlated with the degree of airway obstruction and the number of exacerbations during the previous year. COPD patients categorized as frequent exacerbators were enriched with the bacterial genera *Pseudomonas* which, in turn, was positively associated with the severity of airflow limitation and the prior year’s exacerbation history. The associative relationships of the sputum microbiome with the severity of the disease emphasize the relevance of the interaction between the respiratory microbiota and systemic inflammation.

## 1. Introduction

Chronic obstructive pulmonary disease (COPD), a syndrome characterized by airflow obstruction and chronic inflammation, is considered to be a heterogeneous condition encompassing various phenotypes and endotypes [1]. Widely accepted COPD clinical phenotypes include chronic bronchitis, emphysema, asthma-COPD overlap (ACO), and frequent exacerbators [2]; α-1 antitrypsin deficiency and eosinophilic COPD are among the best-described endotypes [3]. This wide variety of conditions is influenced by both the genotype and environmental factors, which contribute to the complex and diverse pathophysiological mechanisms underlying COPD.

Bacteria are thought to play an important role in COPD pathogenesis, and increasing evidence has shed some light on the understanding of the complex microbial communities present in the lung [4]. The microbiome of the respiratory tract of COPD patients differs from that of healthy subjects [5]. In addition, recent data have shown that the airway microbiome is associated with different COPD inflammatory phenotypes, having the potential to influence the pathogenetic processes underlying chronic inflammation [6,7,8,9,10]. Moreover, perturbations to the airway microbiota have been previously associated with disease severity [11,12], frequency of exacerbations [6,11,13], and medication status [14], and therefore may be used to establish prognosis [15]. However, despite some recent advances regarding associations between the respiratory microbiome and airway inflammatory events occurring in COPD [16], the link between the former and chronic systemic inflammation remains incompletely understood.

In this context, the present study addresses this relationship in two of the most widely accepted phenotypes and treatable traits in COPD, patients with peripheral eosinophilia and those considered as frequent exacerbators. We sought to (1) explore the circulating levels of pro-inflammatory markers and selected eosinophilic-related gene transcripts in stable COPD patients with different clinical and biological conditions and (2), examine their association with the relative abundance of respiratory bacterial genera observed in their spontaneous sputum.

## 2. Results

### 2.1. Demographic and Clinical Features of Participants

Seventy-two stable COPD patients, including 64 males and 8 females, were finally recruited. The average age at inclusion was 67.9 years and all degrees of disease severity were covered. The main demographic and clinical characteristics of these patients are summarized in Table 1.

To explore the potential relationship between microbiome composition and circulating inflammatory marker levels with different clinical conditions, COPD patients were divided into the previously mentioned subgroups, resulting in 52 IE and 20 FE, as well as 57 Non-EOS and 15 EOS. No significant differences in age and BMI were detected between the different subgroup pairs. However, and as presumably expected, FE patients showed a significant impairment in their lung function compared to IE (Table 1), as reflected in their GOLD stages. The severity classification also displayed some differences between Non-EOS and EOS patients, with a higher percentage of GOLD 4 in the former.

### 2.2. All Patients: Blood Inflammatory Mediators and Microbiome Interactions

**Figure 1:**
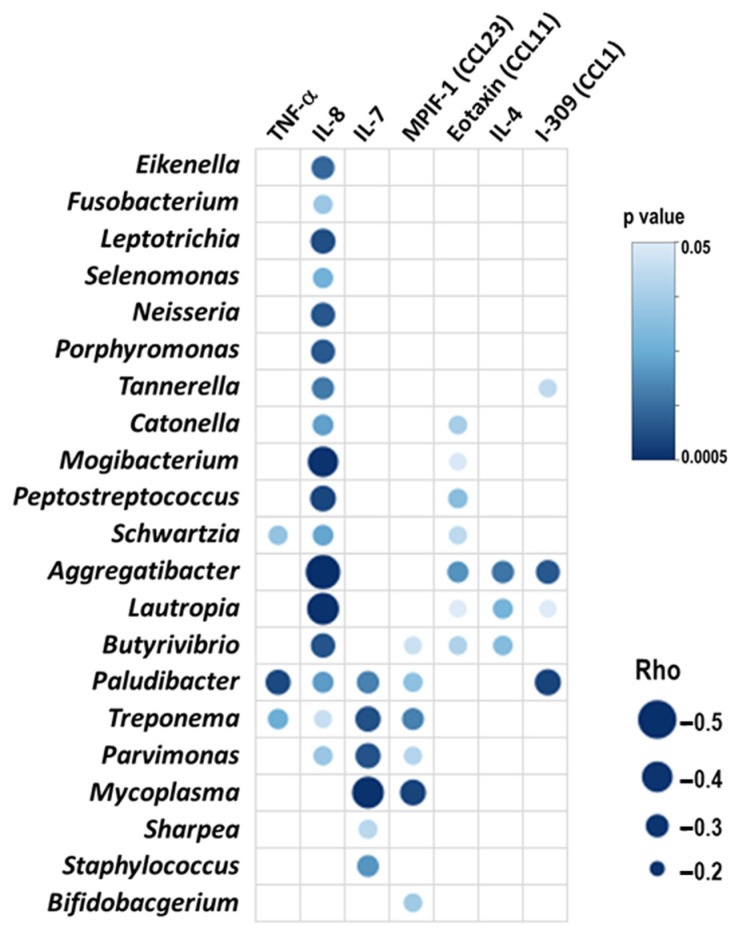
Most representative significant negative (blue) correlations between blood inflammatory markers and the relative abundance of bacterial genera. Bubble size represents the Spearman’s Rank correlation coefficient and the intensity of bubble color represents the *p* value.

To investigate how the respiratory microbiome was related to systemic inflammation, an ‘all-against-all’ correlation analysis between the relative abundance of bacterial genera and a panel of 58 selected blood inflammatory mediators was employed. The most remarkable finding was the negative association exhibited by IL-8 with 17 bacterial genera (Figure 1). Notably, the relative abundance of four of the bacterial genera (*Aggregatibacter*, *Butyrivibrio*, *Treponema,* and *Parvimonas*) associated with the levels of this cytokine appears also to be positively but mildly related to lung function and inversely to the number of exacerbations during the previous year, and both expressing better lung health (Table 2). Other inflammatory mediators showed mixed relationships with bacterial genera (Figure 1).

### 2.3. Blood Inflammatory Mediators and Host–Microbiome Interactions in the FE Phenotype

**Figure 2:**
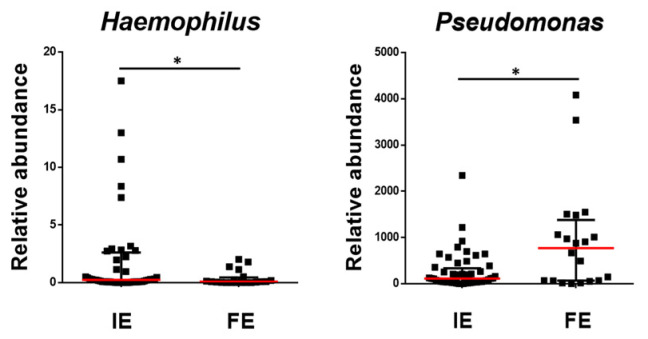
Relative abundance of bacterial genera with significant differences between IE and FE COPD patients. Data are presented as individual data points with median as a red line and IQR as black lines where * is *p* < 0.05.

**Figure 3:**
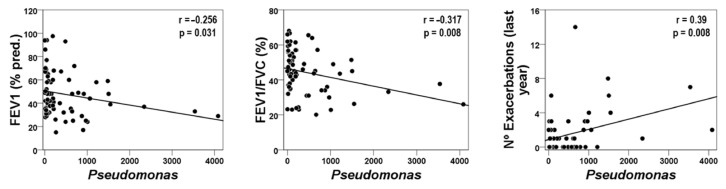
Relationships between the relative abundance of *Pseudomonas* with lung function and the number of exacerbations during the previous year.

Relative abundances of microbial genera were also analyzed according to exacerbation phenotypes. FE patients presented a significant reduction in *Haemophilus*, whereas *Pseudomonas* were more abundant in this group (Figure 2), also showing an inverse relationship between these two microorganisms (Rho = 0.292, *p* < 0.05). None of these potentially pathogenic genera, however, showed any significant associations with the circulating inflammatory mediators nor with any of the eosinophilic-related gene transcripts. Nonetheless, it is worth noting that the acute phase protein CRP showed a clear tendency to be higher in FE patients as compared to IE (9.76 ± 11.6 vs. 9.42 ± 25.58 μg/mL, respectively, *p* = 0.058). Interestingly, the relative abundance of *Pseudomonas* was negatively correlated with some lung function variables (i.e., FEV_1_ and FEV_1_/FVC) and was positively associated with the number of exacerbations during the previous year (Figure 3).

### 2.4. Blood Inflammatory Mediators and Host–Microbiome Interactions in the EOS Pheno/Endotype

**Figure 4:**
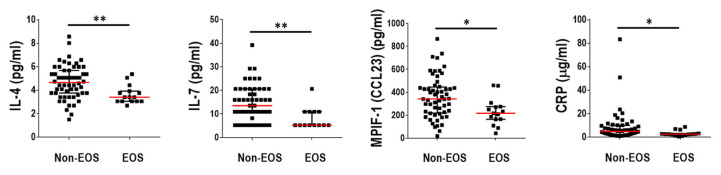
Protein levels of circulating inflammatory markers with significant differences between Non-EOS and EOS patients. Data are presented as individual data points with median as a red line and IQR as black lines. *, *p* < 0.05: **, *p* < 0.01.

**Figure 5:**
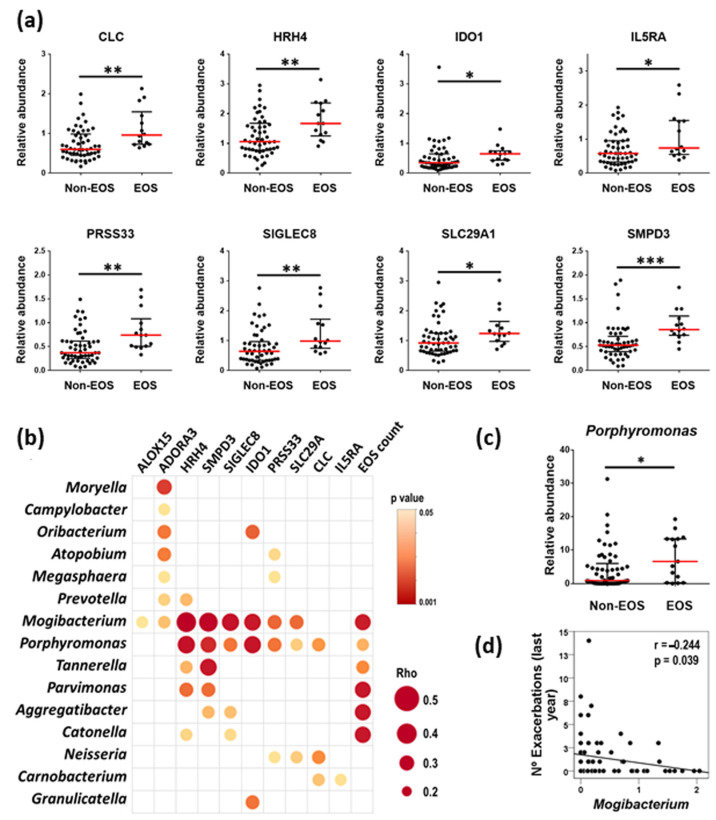
(**a**) Relative expression of eight eosinophil-related gene transcripts with significant differences between Non-EOS and EOS patients. Data are presented as individual data points with median (red line) and IQR (black lines). (**b**) Most representative direct (red) correlations between the level of eosinophilic-related gene transcripts and blood eosinophil counts with the relative abundance of bacterial genera. Bubble size represents the correlation coefficient and the intensity of bubble color represents the *p* value. (**c**) Relative abundance of *Porphyromonas* in EOS and Non-EOS COPD patients. (**d**) Spearman’s Rank correlation of relative abundance of *Mogibacterium* with the number of exacerbations during the previous year. Significances: *, *p* < 0.05; **, *p* < 0.01; ***, *p* < 0.001.

Even though most of the measured blood inflammatory markers did not discriminate EOS from Non-EOS patients, four of them showed significantly lower levels in the former: CRP, IL-4, IL-7, and MPIF-1 (Figure 4). Blood eosinophil counts and the expression levels of 10 eosinophil-related genes [17] were used in turn to analyze the association of eosinophilic inflammation with the respiratory microbiome composition. Expression levels of most of the selected eosinophil marker genes were significantly higher in EOS than in Non-EOS patients (Figure 5a) and showed strong positive correlations with blood eosinophil counts (Table 3). Both, eosinophil counts and most eosinophil-related gene transcripts analyzed showed positive correlations with the relative abundance of several bacterial genera, with particular note for *Porphyromonas* and *Mogibacterium* (Figure 5b). Moreover, the relative abundance of *Porphyromonas* was significantly higher in this subgroup of COPD patients (Figure 5c). *Mogibacterium,* on the other hand, showed an inverse association with the prior year’s exacerbation history (Figure 5d).

## 3. Discussion

The main findings of the present study are the direct relationships observed between the respiratory microbiome with the systemic inflammatory pattern and the clinical severity of the disease. Indeed, on the one hand, the relative abundance of several bacterial genera correlated with the plasma level of several proinflammatory markers, with special note for IL-8. On the other, the categorization of patients based on exacerbation frequency showed a predominance of *Pseudomonas* in the sputum of FE. Moreover, the relative abundance of this microorganism was positively associated with a decline in lung function and the number of exacerbations over the previous year. Finally, when patients were categorized by the blood eosinophilic inflammatory pattern, several bacterial genera, mainly *Porhyromonas* and *Mogibacterium*, showed broad associations with eosinophilic markers. Importantly, part of those bacterial genera associated with either circulating IL-8 levels or some eosinophilic markers also showed a positive association with lung function and a negative one with previous exacerbations (i.e., with a more preserved clinical status). 

### 3.1. Microbiome and Inflammation in COPD

IL-8 has previously been identified as an inflammatory biomarker related to systemic inflammation in COPD patients [18], and although their levels are higher at COPD stability compared with controls, the magnitude of the IL-8 increase was even more remarkable during acute exacerbations, mainly in those patients with the FE phenotype [19,20]. The results of the current study showed an inverse association between IL-8 levels and the relative abundance of several bacterial genera in the sputum of stable COPD patients. Furthermore, a subgroup of these genera (including *Aggregatibacter*, *Butyrivibrio*, *Parvimonas,* and *Treponema*) also showed a positive correlation with lung function parameters and a negative correlation with the number of exacerbations during the previous year, which together can be considered as an expression of a better lung health status. Altogether, these results may suggest that the relative abundance of these bacterial genera at stability may play a protective role against disease impairment through the modulation of the systemic inflammatory status. An alternative explanation would be that lower circulating levels of IL-8 may favor a shift in the microbial composition present in the respiratory system, favoring the proliferation of a relatively beneficial microbiota that may contribute to attenuating lung disease progression. However, although the present results highlight multiple associations between the respiratory microbiome and the profile of the immune response, they do not allow us to establish causality.

### 3.2. The FE Phenotype

COPD is characterized by a progressive impairment of lung function in most patients, a deterioration that becomes worsened by repeated episodes of acute exacerbations [21]. Moreover, the FE phenotype has been associated with an even more rapid disease progression and higher rates of hospitalization and mortality [21]. Exacerbations, typically caused by respiratory tract infections [21], are accompanied by increased airway and systemic inflammation [21], as well as changes in the respiratory microbiome [6,7]. Variability in microbiome composition, however, has also been described as clinical stability and may probably differ across different COPD phenotypes [6,11,12,13,22]. In the present investigation, the association of bacterial microbiota with blood inflammatory markers was analyzed in FE and IE patients during clinical stability. In this regard, the former group showed a significantly higher relative abundance of *Pseudomonas* but lower abundance of *Haemophilus* as compared to the latter. Furthermore, a significant inverse relationship was seen between these two potentially pathogenic microorganisms, suggesting a competitive behavior between them. The *Pseudomonas* level, in addition, was directly related to a more severe disease since it was associated with the impairment of lung function and a higher number of previous exacerbations. Several microbe studies have shown that *Haemophilus* and *Pseudomonas* play a significant role in the pathogenesis and progression of COPD [23], and weaker evidence suggests that Haemophilus may also be associated with more severe airway inflammation [24]. The present results, however, support an anti-inflammatory protecting role for the Haemophilus genus, although additional research will be needed to corroborate this observation.

In line with the present findings, a longitudinal study carried out by Jacobs and cols. Ref. [25] identified negative associations between *Pseudomonas* and *Haemophilus* either at stability or during exacerbations in COPD patients. Moreover, interspecific competition between *Haemophilus* and *Pseudomonas* has also been described in chronic bacterial lung infections present in non-cystic fibrosis bronchiectasis. Moreover, patients with a predominance of *Pseudomonas* infections showed an accelerated decline in their lung function and more frequent respiratory exacerbations [26].

### 3.3. The EOS Phenotype/Endotype

Recent studies have revealed that COPD patients with higher eosinophilic airway inflammation show a distinct structure of bacterial microbiota as compared with either those patients with lower levels of this inflammatory profile or healthy controls [9,11,27]. Moreover, microbiome diversity has been associated with low eosinophil count in peripheral blood [9], and although evidence on the relationship between bronchial and systemic eosinophilia remains controversial, a mounting body of evidence has demonstrated a positive correlation between blood and sputum eosinophil counts [28,29,30]. Subsequently, blood eosinophil counts have emerged as a simple, sensitive, and easily obtained biomarker that can be used in clinical practice. In this study, we sought to explore the associations of respiratory bacterial microbiota with blood eosinophilic inflammation. For this purpose, eosinophilic patients were categorized based on blood percentages of eosinophils and neutrophils. In addition to blood eosinophil proportion, a group of eosinophil-related gene transcripts was used as eosinophilic markers [17]. On the other hand, patients with a low percentage of blood neutrophils were used to reduce biases due to the inflammatory influence of these cells (as it has been previously used to categorize airway inflammatory patterns) [31].

Upregulation of eosinophilic-related gene transcripts in EOS patients and their high correlation with blood eosinophil counts render these genes as reliable surrogate markers to assess differences between EOS and Non-EOS COPD patients. The present study shows multiple positive interactions between eosinophil-related markers and some bacterial genera (with special note for *Porphyromonas* and *Mogibacterium*), suggesting an interplay of airway bacterial microbiota with systemic eosinophilic inflammation. Moreover, the relative abundance of *Mogibacterium* was inversely related to the number of exacerbations over the previous year. These findings partially differ from most published research, which shows a greater risk of exacerbation in patients with high blood eosinophil levels [32,33,34]. However, a deep analysis of two well-phenotyped COPD cohorts (COPDGene and ECLIPSE), revealed that the association of an increased exacerbation risk associated with elevated eosinophil counts was driven by the history of frequent exacerbations [17]. Moreover, a recent report by Miravitlles et al. [35] does not support the use of blood eosinophil count as a reliable marker of the risk of exacerbation in stable COPD patients. These authors reported that patients with the lowest blood eosinophil levels presented the highest frequency of acute episodes during the previous year. Some of these discrepancies may be due to variability in oscillations in eosinophil counts over time and the relative lack of an optimal cut-off for it (either absolute or percentage), which do not allow a reliable and uniform stratification of patients. Larger studies with extended follow-up periods will be needed to provide more insight into this topic.

As for the rest of the biomarkers studied, we found no significant differences between groups except for a reduction in four inflammatory factors, including the acute phase protein CRP, in EOS patients. Lower CRP levels in patients with high eosinophil counts have also been described by previous work [36]. Elevated CRP levels, on the other hand, have been associated with the severity of airway obstruction [37], an elevated risk of having exacerbations [38], a poor short-term outcome in patients admitted for these acute episodes [39], and higher mortality [40].

The present study has several potential limitations. First of all, its cross-sectional nature does not allow us to make causal inferences about the impact of the microbiome on systemic inflammation and clinical outcomes. Another limitation is that we only analyzed the respiratory microbiome of stable COPD patients, and therefore, we could not extend it to the analysis of the potential associations between respiratory microbiota and biological markers in healthy individuals. Additionally, stratification by clinical phenotypes implies a substantial reduction in the number of patients per group limiting the statistical power of the analyses and the conclusiveness of the results. Moreover, the predominance of male subjects in our cohort may introduce some bias due to sex physiological differences. Finally, some of the bacterial genera were barely detected in a significant number of patients, so we were unable to assess their association with inflammation and clinical outcomes in the present cohort.

In conclusion, the present investigation shows that some changes in the bacterial composition of the pulmonary microbiota relate to the systemic inflammatory response and are associated with clinical outcomes. These relationships may be complex and bidirectional. Thus, further studies involving larger cohorts of patients, longitudinal designs, and control for potential confounding factors are necessary to examine the interplay between microbiota and systemic inflammation in more depth.

## 4. Materials and Methods

### 4.1. Study Design and Population

This investigation is embedded within the BIOMEPOC project, a prospective controlled multicenter study whose details have been published elsewhere [11,41]. For the current study, stable COPD patients were sequentially recruited from five teaching hospitals, and ethics approval was granted locally at each site by Institutional ethics committees. The investigation was conducted in accordance with the Declaration of Helsinki and informed written consent was obtained from all patients. They were subsequently subdivided by their history of exacerbations [Infrequent exacerbators (IE) vs. Frequent exacerbators (FE)] and by their distinct inflammatory profiles [Non-Eosinophilic (Non-EOS) vs. Eosinophilic (EOS)]. The FE condition was defined as the presence of ≥2 exacerbations over the year preceding the patient’s recruitment [42]. EOS patients were defined as those presenting ≥3% blood eosinophils with <60% circulating neutrophils. This stratification was adapted from previous classifications based on the pattern of airway inflammation in sputum samples [31].

### 4.2. Samples Processing

Samples from each participant included spontaneous sputum, plasma, and whole blood. Spontaneous sputum was collected and processed within 60 min on the day of the visit. Subsequently, sputum quality was assessed as previously described [11] and samples were frozen until processing. Blood samples in turn were collected by venipuncture and placed into K_3_-EDTA tubes for plasma obtention and in Tempus tubes (Thermo Fisher Scientific, Waltham, MA, USA) for RNA isolation. K_3_-EDTA tubes were centrifuged at 1500× *g* for 15 min at 4 °C, and plasma supernatants were stored at −80 °C until further analyses. Total RNA was purified using the Tempus™ Spin RNA Isolation Kit following the provider’s recommendations (Thermo Fisher Scientific).

### 4.3. 16S rRNA Gene Sequencing Analysis

16S rRNA gene was amplified following the 16S Metagenomic Sequencing Library Preparation Illumina protocol (Part # 15044223 Rev. A, Illumina, CA, USA). The Quantitative Insights Into Microbial Ecology (QIIME) pipeline 1.9.0 was used for sequence processing to obtain taxonomic information. Detailed procedures of DNA extraction, PCR, and library preparation as well as analysis of the microbiome profile of the present cohort have been previously published by our group [11].

### 4.4. Measurement of Systemic Inflammatory Mediators

Inflammatory protein profiles were measured in plasma using three magnetic bead-based multiplex immunoassays, namely, Bio-Plex Pro Human Chemokine Panel (40-Plex #171AK99MR2), Bio-Plex Pro Human Cytokine Panel (27-Plex #M500KCAF0Y) and Bio-Plex Pro Human Acute Phase Panel (4-Plex #171A4C09M) following the manufacturer’s specifications (Bio-Rad, Hercules, CA, USA). Briefly, magnetic beads coated with specific antibodies were incubated with the samples for 30 min. After this incubation, the beads were washed using a magnetic plate washer (Bio-Plex Pro Wash Station, Bio-Rad), and then reincubated with biotinylated detection antibodies for 60 min. Then, the beads were washed and incubated with streptavidin-PE for 10 more minutes. The beads were then washed, resuspended in the assay buffer, and run on the BioPlex-200 instrument (Bio-Rad) to measure the analytes. All samples were performed in only one setting. It is worth noting that due to some redundancy between the kits, only 58 immune-soluble constituents were analyzed for each sample. The list of names, acronyms, and official gene symbols for all factors measured are described in Table S1.

### 4.5. RT-qPCR Assays

First-strand cDNA was generated using oligo (dT)_12-18_ and Superscript III reverse transcriptase as per the manufacturer’s protocol (Thermo Fisher Scientific). Quantitative PCR of selected genes was carried out using TaqMan Gene Expression Assays products (Table S2) in an ABI PRISM 7900 HT Sequence Detection System (Applied Biosystems, Waltham, MA, USA). Standard TaqMan cycling conditions were used, and all reactions were performed in triplicate. Gene expression levels were normalized to the housekeeping genes GAPDH (Glyceraldehyde-3-phosphate dehydrogenase) and ACTB (Beta-actin), and the relative gene expression analysis was done using the comparative method (Delta-Delta C(T)) [43] by ExpressionSuite Software v1.3.

### 4.6. Statistical Analyses

Descriptive statistics: Continuous variables are expressed as mean and standard deviations (SD) values if normally distributed or as medians and interquartile ranges if not normally distributed. Categorical variables are presented as frequencies and percentages. Comparisons between study groups were done using independent samples T-tests or Mann–Whitney *U* tests for normally and not normally distributed variables, respectively. Spearman Rank correlation coefficients were computed to assess associations between the relative abundance of bacterial genera with the plasma concentration of inflammatory and eosinophilic markers, as well as with the clinical variables. Statistical tests used in the study were two-sided, and a *p* value of ≤0.05 was considered as statistically significant. Statistical analyses were performed using the SPSS statistical software package version 23 (SPSS Inc., Chicago, IL, USA), and GraphPad Prism6 (Dotmatics, Boston, MA, USA) and Cytoscape software (version 3.8.0, Seattle, WA, USA) were used for chart production.

## Figures and Tables

**Table 1:** Clinical characteristics of patients.

	All (N = 72)	IE COPD (N = 52)	FE COPD (N = 20)	Non-EOS COPD (N = 57)	EOS COPD (N = 15)
Age, years median (IQR)	70 (62–73)	69 (62–73)	70 (66–74)	70 (62–73)	69 (62–74)
Sex (male), n (%)	64 (89)	46 (89)	18 (90)	50 (88)	14 (93)
BMI, kg/m^2^ median (IQR)	27.4 (24.2–30.1)	27.4 (25.1–29.9)	27.5 (23.5–30.7)	27.8 (24.1–30.5)	27.3 (24.7–28.4)
FEV_1_, (% pred.) median (IQR)	44 (33–60)	48 (35–67)	34 (26–47) **	43 (32–60)	48 (35–67)
FEV_1_/FVC, (%) median (IQR)	44 (35–55)	46 (37–57)	35 (30–45) **	45 (34–54.3)	43 (37–56)
DLco, (% pred.) median (IQR)	51 (40–68)	51 (45–71)	40 (32–60) *	49 (40–66)	65 (40–82)
Nº of Exacerbations in the previous year, median (IQR)	1 (0–2)	0 (0–1)	3 (2–6) ***	1 (0–2)	0 (0–1)
Blood leucocytes (cells/nL), median (IQR)	8 (6.7–9.3)	7.6 (6.7–8.7)	8.4 (7–10.6)	8 (6.7–10)	7.1 (6.5–8.5)
Blood eosinophils (cells/nL), median (IQR)	0.2 (0.1–0.3)	0.2 (0.1–0.3)	0.2 (0.1–0.3)	0.2 (0.1–0.2)	0.3 (0.2–0.3) **^‡‡‡^**
Blood neutrophils (cells/nL), median (IQR)	4.6 (3.9–6.1)	4.5 (3.8–5.3)	5.7 (4–4.8)	4.8 (4.1–6.3)	3.8 (3.4–4.6) ^‡‡‡^
GOLD stages, n (%)					
1	6 (8)	6 (12)	0 (0)	4 (7)	2 (13)
2	20 (28)	17 (32)	3 (15)	15 (26)	5 (33)
3	35 (49)	26 (50)	9 (45)	28 (49)	7 (47)
4	11 (15)	3 (6)	8 (40) ***	10 (18)	1 (7)

Notes: Data are presented as median (IQR) for continuous variables and n (percentage) for categorical variables. *, *p* < 0.05; **, *p* ≤ 0.01; ***, *p* ≤ 0.001 for IE vs. FE COPD patients. ^‡‡‡^, *p* ≤ 0.001 for Non-EOS vs. EOS COPD patients. Abbreviations: COPD, Chronic Obstructive Pulmonary Disease; IE, infrequent exacerbators; FE, frequent exacerbators; Non-EOS, non-eosinophilic; EOS, eosinophilic; BMI, body mass index; FEV_1_, forced expiratory volume in 1 s; FVC, forced vital capacity; DLco, diffusing capacity of the lungs for carbon monoxide; GOLD, Global initiative for obstructive lung disease.

**Table 2:** Bacterial genera and clinical variables with significant correlations.

	FEV_1_ (% Pred)	DLco (% Pred) ^‡^	Nº EXACERBATIONS (Previous Year)
*Aggregatibacter*	0.252 *	0.282 *	−0.364 **
*Butyrivibrio*	0.248 *	0.320 *	−0.311 **
*Treponema*	0.436 ***	0.384 **	−0.305 **
*Parvimonas*	0.250 *	0.335 *	-

Notes: Spearman’s correlation coefficients. *, *p* < 0.05; **, *p* < 0.01; and ***, *p* ≤ 0.001. ^‡^ Data missing for 20 patients. Abbreviations: FEV_1_, forced expiratory volume in 1 s; DLco, diffusing capacity of the lungs for carbon monoxide.

**Table 3:** Relationship between Eosinophilic-related gene transcripts and blood eosinophil counts.

	EOSINOPHIL COUNTS
ADORA3	0.517 ***
ALOX15	0.572 ***
CLC	0.710 ***
HRH4	0.547 ***
IDO1	0.508 ***
IL5RA	0.513 ***
PRSS33	0.703 ***
SIGLEC8	0.712 ***
SLC29A1	0.632 ***
SMPD3	0.618 ***

Notes: Spearman’s correlation coefficient. ***, *p* < 0.001. Abbreviations: ADORA3: Adenosine A3 receptor; ALOX15: Arachidonate 15-Lipoxygenase; CLC: Charcot–Leyden crystal galectin; HRH4: Histamine Receptor H4; IDO1: Indoleamine 2,3-Dioxygenase 1; IL5RA: Interleukin 5 receptor subunit Alpha; PRSS33: Protease, Serine 33; SIGLEC8: Sialic acid-binding Ig-like Lectin 8; SLC29A1: Solute carrier family 29 member 1; SMPD3: Sphingomyelin Phosphodiesterase 3.

## Data Availability

The raw data supporting the conclusions of this article will be made available by the authors on request.

## Supplementary Materials

The following supporting information can be downloaded at: https://www.mdpi.com/article/10.3390/ijms25158467/s1.

## Appendix A

Members of the BIOMEPOC group (alphabetical order): Mireia Admetlló ^1,2^, Alvar Agustí ^2,3^, Carlos Alvarez-Martínez ^4^, Esther Barreiro ^1,2^, Oswaldo Antonio Caguana ^1^, Carme Casadevall ^1,2^, Ferran Casals ^5^, Robert Castelo ^5^, Ady Castro-Acosta ^4^, Rocío Córdova ^6^, Borja G Cosío ^2,6^, Rosa Faner ^2,3^, Laura I Furlong ^5,7^, Marian García ^8^, José G. González-García ^1^, Carmen Hernández-Carcereny ^2,3^, José Luis López-Campos ^2,9^, Eduardo Márquez ^2,9^, Eduard Monsó ^8^, Concepción Montón ^8^, Miren Josune Ormaza ^8^, Alexandre Palou ^6^, Sergi Pascual ^1^, Germán Peces-Barba ^2,10^, Pau Puigdevall ^5^, Diego A. Rodríguez-Chiaradia ^1,2^, Ferran Sanz ^5,7^, Luis Seijó ^11^, Montserrat Torà ^5,12^, Yolanda Torralba ^3^, Carles Vilaplana ^13^.

1. Hospital del Mar Research Institute. Respiratory Medicine Department, *Hospital del Mar*. MELIS Dept., *Universitat Pompeu Fabra* (UPF). BRN. Barcelona. Spain.
2. *Centro de Investigación Biomédica en Red*, *Área de Enfermedades Respiratorias* (CIBERES), *Instituto de Salud Carlos III*. Madrid. Spain.
3. *Servei de Pneumologia* (*Institut Clínic de Respiratori*), *Hospital Clínic*-*Fundació Clínic per la Recerca Biomèdica*, *Universitat de Barcelona*. Barcelona. Spain.
4. *Servicio de Neumología*, *Hospital 12 de Octubre*. Madrid. Spain.
5. Dept. MELIS, *Universitat Pompeu Fabra*. Barcelona. Spain.
6. *Servicio de Neumología*, *Hospital Son Espases*-*Instituto de Investigación Sanitaria de Palma* (IdISBa), *Universitat de les Illes Balears*. Palma de Mallorca. Spain.
7. *Hospital del Mar Research Institute*. Barcelona. Spain.
8. *Servicio de Neumología*, *Consorci Sanitari Parc Taulí*, *Universitat Autònoma de Barcelona*. Sabadell. Spain.
9. *Unidad Médico-Quirúrgica de Enfermedades Respiratorias*, *Hospital Universitario Virgen del Rocío*, *Universidad de Sevilla*. Sevilla. Spain.
10. *Servicio de Neumología*, *Fundación Jiménez Díaz*, *Universidad Autónoma de Madrid*. Madrid. Spain
11. *Servicio de Neumología*, *Fundación Jiménez Díaz*, *Universidad Autónoma de Madrid*. *Clínica Universidad de Navarra*. Madrid. Spain.
12. *Universitat Autònoma de Barcelona*. Barcelona. Spain.
13. *Laboratori de Referència de Catalunya*. *El Prat de Llobregat*. Spain.
