# Supplementary material for: Relationship between Respiratory Microbiome and Systemic Inflammatory Markers in COPD: A Pilot Study
Source: Int J Mol Sci. 2024 Aug 2;25(15):8467. doi: 10.3390/ijms25158467 (PMC11313397; doi:10.3390/ijms25158467)
Supplement: Supplementary file 1 [file ijms-25-08467-s001.zip › ijms-3128358-supplementary.pdf]

Table S1

Cytokines, chemokines, growth factors and acute phase proteins measured in the current study

Protein abbreviations	Gene ID	Protein name
IL-1β	IL1B	Interleukin-1β
IL-1RA	IL1RN	Interleukin-1 receptor antagonist
IL-2	IL2	Interleukin-2
IL-4	IL4	Interleukin-4
IL-5	IL5	Interleukin-5
IL-6	IL6	Interleukin6
IL-7	IL7	Interleukin-7
CXCL8 / IL-8	CXCL8	C-X-C motif chemokine ligand 8
IL-9	IL9	Interleukin-9
IL-10	IL10	Interleukin10
IL-12p70	IL12p70	Interleukin-12
IL-13	IL13	Interleukin-13
IL-15	IL15	Interleukin-15
IL-16	IL16	interleukin 16
IL-17	IL17	Interleukin-17
IFN-γ	IFNG	Interferon gamma
TNF-α	TNF	Tumor necrosis factor-alpha
FGF2	FGF2	Fibroblast growth factor 2, Basic fibroblast growth factor
G-CSF	CSF3	Granulocyte colony stimulating factor, Colony Stimulating Factor 3
GM-CSF	CSF2	Granulocyte-macrophage colony stimulating factor, Colony Stimulating Factor 2
PDGF	PDGFB	Platelet derived growth factor subunit B
VEGF	VEGFA	Vascular endothelial growth factor
MIF	MIF	Macrophage migration inhibitory factor
CCL1 / I-309	CCL1	T lymphocyte-secreted protein I-309
CCL2 / MCP1	CCL2	C-C Motif chemokine ligand 2
CCL3 / MIP-1α	CCL3	Macrophage inflammatory protein-1 alpha, C-C Motif chemokine ligand 3
CCL4 / MIP1β	CCL4	C-C Motif chemokine ligand 4, Macrophage inflammatory protein 1-Beta,
CCL5 / RANTES	CCL5	C-C Motif chemokine ligand 5
CCL7 / MCP-3	CCL7	monocyte chemotactic protein-3
CCL8 / MCP-2	CCL8	monocyte chemotactic protein-2
CCL11 /Eotaxin	CCL11	Eosinophil chemotactic protein
CCL13 / MCP-4	CCL13	monocyte chemotactic protein-4
CCL15 / MIP-1d	CCL15	macrophage inflammatory protein-1 delta
CCL17 / TARC	CCL17	Thymus and activation-regulated chemokine
CCL19 / MIP-3b	CCL19	Macrophage inflammatory protein 3 beta
CCL20 / MIP-3a	CCL20	Macrophage inflammatory protein 3 alpha
CCL21 / 6Ckine	CCL21	C-C motif chemokine 21
CCL22 / MDC	CCL22	Macrophage-derived chemokine
CCL23 / MPIF-1	CCL23	Myeloid progenitor inhibitory factor 1
CCL24 /Eotaxin-2	CCL24	Eosinophil chemotactic protein 2
CCL25 / TECK	CCL25	Thymus-expressed chemokine
CCL26 / Eotaxin-3	CCL26	Macrophage inflammatory protein 4-alpha
CCL27 / CTACK	CCL27	cutaneous T cell-attracting chemokine
CXCL1 / Gro-alpha	CXCL1	Growth-regulated alpha protein
CXCL2 / Gro-beta	CXCL2	Growth-regulated protein beta
CXCL5 / ENA-78	CXCL5	Epithelial-derived neutrophil-activating protein 78
CXCL6 / GCP-2	CXCL6	Granulocyte chemotactic protein 2
CXCL9 / MIG	MIG	monokine induced by interferon gamma
CXCL10 / IP-10	CXCL10	C-X-C motif chemokine ligand 8, Interferon gamma-induced protein 10
CXCL11 / I-TAC	CXCL11	Interferon-inducible T-cell alpha chemoattractant
CXCL12 / SDF1a+b	CXCL12	Stromal cell-derived factor 1
CXCL13 / BCA-1	CXCL13	B cell-attracting chemokine 1
CXCL16 / SCYB16	CXCL16	Small-inducible cytokine B16
CX3CL1 / Fractalkine	CX3CL1	Fractalkine
Alpha-2-M	A2M	Alpha-2-macroglobulin
Haptoglobin	HP	Haptoglobin
C-Reactive Protein	CRP	C-Reactive Protein
SAP	APCS	Serum Amyloid P-component

Table S2

TaqMan Gene Expression Assays used for gene expression analyses

Gene Symbol	Gene Name	TaqMan Assay ID
**Selected Genes**		
**ALOX15**	Arachidonate 15-Lipoxygenase	Hs00993765_g1
**CLC**	Charcot-Leyden crystal galectin	Hs00171342_m1
**HRH4**	Histamine Receptor H4	Hs00222094_m1
**IDO1**	indoleamine 2,3-dioxygenase 1	Hs00984148_m1
**IL5RA**	Interleukin 5 receptor subunit alpha	Hs00602482_m1
**PRSS33**	Protease, serine 33	Hs00541732_m1
**SIGLEC8**	Sialic acid-binding Ig-like lectin 8	Hs00274289_m1
**SLC29A1**	Solute carrier family 29 member 1	Hs01085706_m1
**SMPD3**	Sphingomyelin phosphodiesterase 3	Hs00920354_m1
**ADORA3**	Adenosine A3 receptor	Hs00181232_m1
**Housekeeping genes**		
**ACTB**	Actin beta	Hs99999903_m1
**GAPDH**	glyceraldehyde-3-phosphate dehydrogenase	Hs99999905_m1
